# Sex-driven neighborhood effects on herbivory in the dioecious Mediterranean palm *Chamaerops* *humilis* L.

**DOI:** 10.1007/s00442-023-05457-z

**Published:** 2023-10-05

**Authors:** Raquel Muñoz-Gallego, Thorsten Wiegand, Anna Traveset, Jose M. Fedriani

**Affiliations:** 1 Global Change Research Group, Mediterranean Institute of Advanced Studies (IMCDEA, CSIC-UIB), C/ Miquel Marquès, 21, 07190 Esporles, Balearic Islands Spain; 2 Department of Ecological Modelling, Helmholtz Centre for Environmental Research GmbH-UFZ, Leipzig, Germany; 3 German Centre for Integrative Biodiversity Research (iDiv) Halle-Jena-Leipzig, Leipzig, Germany; 4 Desertification Research Centre (CIDE, CSIC), Crta. Moncada-Náquera, Km 4.5, 46113 Moncada, Valencia Spain; 5 Doñana Biological Station (EBD, CSIC), C/Americo Vespucio s/n, 41092 Seville, Spain

**Keywords:** Distance- and density-dependence, Florivory, Folivory, Goat, *Paysandisiaarchon*, Spatial point pattern analyses

## Abstract

Although it is well recognized that the strength of plant–herbivore interactions can vary with the plant sex, the distance, and the density of conspecific neighbors, no study has yet assessed their combined influence. Here, we filled this knowledge gap by focusing on the dioecious palm *Chamaerops* *humilis* L., and its two main herbivores, the invasive moth *Paysandisia* *archon* Burmeister and the feral goat *Capra* *hircu*s L. We evaluated levels and spatial patterns of herbivory, as well as those of plant size and number of inflorescences in two palm populations in Mallorca (Balearic Islands, Spain). Our spatial point pattern analyses revealed that palms not affected by moth herbivory or goat florivory were spatially aggregated, goats fed more strongly upon inflorescences in palms with more neighbors, but they consumed more leaves in isolated palms. Interestingly, we could reveal for the first time that plant sex is a key plant trait modulating neighborhood effects. For instance, whereas aggregated female palms experienced lower intensity of goat florivory than isolated ones, male palms showed the opposite pattern. Palm size and number of inflorescences also showed sex-related differences, suggesting that sexual dimorphism is a key driver of the observed neighborhood effects on herbivory. Our study highlights the importance of considering relevant plant traits such as sex when investigating plant neighborhood effects, calling for further research to fully understand the dynamics governing plant–herbivore interactions in dioecious systems.

**Supplementary Information:**

The online version contains supplementary material available at 10.1007/s00442-023-05457-z.

## Introduction

Around 18,000 angiosperm plant species have a dioecious sexual system (Renner 2014), with individuals that bear either female or male flowers separately within the population. These individuals evolved a sexual dimorphism with divergent attributes, especially in reproduction and defense (Bawa 1980; Barrett and Hough 2012). For example, reproductive traits, such as flower size, number of flowers, nectar secretion, or nutrient content, often differ markedly between sexes (Barrett and Hough 2012; Vega-Frutis et al. 2013; Ndem-Galbert et al. 2021; Calaça et al. 2022). Also to ensure successful reproduction, female plants are thought to invest more in defensive mechanisms than males (Cornelissen and Stiling 2005; Cepeda-Cornejo and Dirzo 2010; Tsuji and Sota 2010; Avila-Sakar and Romanow 2012). There is also empirical evidence for biased sex ratios in dioecious populations, with males being twice as frequent as females (Field et al. 2012; Sinclair et al. 2012).

Biotic interactions, such as facilitation between plants (e.g.,Montesinos et al. 2007; Martín-Forés et al. 2022), plant–pathogen interactions (e.g.,Kaltz and Shykoff 2001; Moritz et al. 2016), or plant–animal interactions (Vega-Frutis et al. 2013), can be influenced by sex-specific plant traits. For example, differences between plant sexes in plant-pollinator interactions (Zhang and He 2017; Calaça et al. 2022; Moquet et al. 2022) and plant–herbivore interactions (Cornelissen and Stiling 2005; Avila-Sakar and Romanow 2012; Moritz et al. 2018; Liu et al. 2021) are well described in the literature. In particular, a review conducted by Cornelissen and Stiling (2005) revealed widespread male-biased herbivory, which was attributed to sexual differences in chemical protection and tissue nutritional quality. However, there is also some evidence for female-biased herbivory (Kabir et al. 2014; Maldonado-López et al. 2014), and even absence of a generalized sex bias in herbivory has been recently suggested (Sargent and McKeough 2022).

The probability and the intensity of herbivory on a given plant can also be determined by traits other than sex, such as ontogeny, height, or nutritional quality (e.g., Miller et al. 2007; Cornelissen et al. 2008; Massad 2013), and by extrinsic factors, such as the density and the distance of conspecific neighbors (Underwood et al. 2014). Higher densities of conspecific neighbors can either attract herbivores (‘resource concentration effect’; Root 1973) or deter them (‘resource dilution effect’; Otway et al. 2005). These trait- and density-related conspecific effects may only operate up to certain distances from the focal plant, resulting in spatially explicit conspecific neighborhood effects (Kim and Underwood 2015; Holík and Janík 2022). While sexual dimorphism is prevalent in dioecious species and has been demonstrated to influence herbivory (Cornelissen and Stiling 2005), the role of plant sex as a driver of neighborhood effects remains unstudied.

Our main goal was to investigate whether and how the effect of distance to and density of conspecific neighbors on herbivory varies between plant sexes. To this end, we studied the dioecious Mediterranean dwarf palm *Chamaerops* *humilis* and its two main herbivores, the invasive moth *Paysandisia* *archon* and the feral goat *Capra* *hircus*. Although the impact of these two herbivores on *C.* *humilis* reproduction (Muñoz-Gallego et al. 2022, 2023) and the spatial ecology of moth attack (Ruiz et al. 2018) have been recently studied, nothing is known about potential differences between palm sexes with respect to neighborhood effects. Specifically, we seek to test the following four hypotheses:

1. The distance and the density of conspecific neighbors influence probability and intensity of herbivory by invasive moths and feral goats on the Mediterranean palm *Chamaerops* *humilis*. Since Ruiz et al. (2018) described contrasting neighborhood effects on moth herbivory across study sites, we also expect some differences between our plots. Moreover, the vegetation patchiness and the density of conspecific neighbors increase herbivory by goats (De Knegt et al. 2007; Chebli et al. 2020) and other ungulates (e.g.,Holík et al. 2021; Murphy and Comita 2021; Holík and Janík 2022). As our target herbivores show divergent phylogeny (insect vs. mammal) and feeding behavior, we also expect that the sign and spatial extent of the neighborhood effects will vary between both herbivorous species.
2. The intensity of herbivory differs between female and male palms due to different reproductive display (i.e., number and nutritional quality of flowers and inflorescences) and different defensive mechanisms between sexes in *C.* *humilis* (Herrera 1989a, b; Fedriani and Delibes 2011; this work). We therefore predict that male palms are preferred by herbivores over female palms (i.e., the male-biased hypothesis; Cornelissen and Stiling 2005).
3. If the previous hypotheses are true, and because the density of conspecific neighbors and plant sex can jointly influence herbivory levels (Bergvall et al. 2006; Kim and Underwood 2015), we predict that neighborhood effects on herbivory will differ between palm sexes.
4. Given that plant traits, such as plant size and flower abundance, which influence herbivory, often differ between female and male plants in dioecious species (Barrett and Hough 2012; Calaça et al. 2022), we also expect sex-specific neighborhood effects on these traits. This analysis may help us better understand the role of plant sex as a driver of neighborhood effects on herbivory (previous hypothesis).

## Methods

### Study system

The Mediterranean dwarf palm *Chamaerops* *humilis* L. (Arecaceae) is a representative species of the Pre-Pliocene paleo-tropical ancestral lineages (Thompson 2005) and the only native palm in the Western Mediterranean region (García-Castaño et al. 2014; Guzmán et al. 2017). It is considered a keystone species in the coastal and the subcoastal ecosystems (Herrera 1989b; Garrote et al. 2022) and, especially, on island systems where trophic resources are often scarce relative to mainland areas (Whittaker and Fernández-Palacios 2007). This dioecious plant blooms during spring (March–May), and male palms earlier than females. Flowers form dense yellow inflorescences of up to 40 cm long and located up to 2 m from the ground level that develop within a single enlarged bract. Male inflorescences bear many more flowers than female ones, and usually produce large amounts of pollen (214,000 grains of pollen on average, Herrera 1989b). Female flowers are tri-ovulate and often secrete nectar. Larval development of the obligate pollinator *Derelomus* *chamaeropis* takes place more often and abundantly within male than female inflorescences (Dufaÿ and Anstett 2004; Jácome-Flores et al. 2018). Moreover, to attract pollinators, male leaves emit a larger amount of volatile compounds than female leaves (Dufaÿ et al. 2003). Sex ratio is not strictly biased, and it can vary spatially (Anstett 1999; Dufaÿ and Anstett 2004; Jácome-Flores et al. 2018). Dwarf palm populations usually show a strongly aggregated spatial distribution at small scales (up to 10 m), both in Mallorca (Muñoz-Gallego et al. 2023) and in southwestern Spain (Jácome-Flores et al. 2016; Garrote et al. 2019).

The invasive Neotropical moth *Paysandisia* *archon* Burmeister (Castniidae, Lepidoptera) is spreading worldwide, having been recorded in more than 10 European countries so far. It is becoming a serious pest of palm trees, especially for *C.* *humilis* (Muñoz-Adalia and Colinas 2020). It was first reported in Mallorca in 2003 (Sarto i Monteys and Aguilar 2005), and its attack on the natural dwarf palm populations of the island is becoming alarming, with up to 40% of the individuals infested in some areas (Ruiz et al. 2018; Muñoz-Gallego et al. 2022). From mid-May to late September, the female moths search for suitable palm crowns to oviposit through chemical and visual cues (Ruschioni et al. 2015; Hamidi and Frérot 2016; Frérot et al. 2017). Annual and biannual larvae feed inside the trunk, triggering the deformation and eventually the stem’s death (Muñoz‐Adalia and Colinas 2020). Spatially explicit analyses conducted by Ruiz et al. (2018) revealed contrasting distance- and density-dependent processes in *C.* *humilis* performance against *P.* *archon* attack depending on the population and the invasion phase. Furthermore, larger palms were more prone to be attacked, but no differences were found between palm sexes.

Feral goats *Capra* *hircus* L. have been described as one of the most dangerous introduced herbivores, especially on islands (Chynoweth et al. 2013; Gizicki et al. 2018; Capó et al. 2022). Their generalist feeding habits involve the consumption of most plant tissues, from vegetative (leaves, stems, and bracts) to reproductive ones (buds, flowers, and developing fruits), having severe direct and indirect effects on plant performance (Gizicki et al. 2018; Muñoz-Gallego et al. 2022). In Mallorca, although the introduction of domestic goats dates back thousands of years (Bover and Alcover 2008), the rural abandonment in the 1960s led to the feralization and the expansion of their populations all over the island (more than 20,000 individuals; Vives and Baraza 2010; Roque 2017). Their impacts on native flora have been well documented in several studies (Cursach et al. 2013; Capó et al. 2021; Muñoz-Gallego et al. 2022) as well as their feeding behavior (Rivera-Sánchez et al. 2015). Specifically, leaves and inflorescences of *C.* *humilis* are intensely consumed by goats, leading to negative effects on pollination and fruit set (Muñoz-Gallego et al. 2022).

### Study plots

Fieldwork was conducted during spring (April–June) of 2019 and 2020 on Mallorca (Balearic Islands, Spain). The climate of the Balearics is typically Mediterranean, characterized by two rainy seasons, a hot dry summer and a soft winter. Most rainfall occurred in October 2019 and April 2020, and there was an extreme drought in June 2019. Annual average temperature was *ca.* 18.5 °C during both study years (data from the *Balearic* *Islands* *Weather* *Network*). We selected two *C.* *humilis* populations (separated by more than 25 km) in which both goats and the invasive moths were present: (1) “Ermita de Betlem (*EB*, hereafter)” (120.82 m × 118.78 m, 39° 44′ 20.5" N 3° 18′ 52.4" E), and (2) “Platja de Formentor (*PF*, hereafter)” (76.36 m × 79.79 m, 39° 55′ 54.9" N 3° 08′ 11.4" E). The former, located in the *Serra* *de* *Llevant*, is a dense palm population with several herbaceous and shrub species, such as *Ampelodesmos* *mauritanicus*, *Asphodelus* *aestivus*, *Smilax* *aspera*, and some scattered olive trees (*Olea* *europaea*), whereas the second is located in the north of *Serra* *de* *Tramuntana* mountain range and consists of a mixed forest of holm oak (*Quercus* *ilex*) and pine trees (*Pinus* *halepensis*) with some shrubs, such as *Pistacia* *lentiscus* and *Erica* *multiflora*. The study areas are characterized by sandy sedimentary soils with sandstone and limestone conglomerates, although *EB* is more lime-filled and rocky (IGME 2023).

### Field sampling

To investigate potential conspecific neighborhood effects on the palm-herbivore interactions, we geo-referenced all *C.* *humilis* individuals in both target populations and monitored them during two consecutive years (2019 and 2020). Overall, we sampled 503 dwarf palms (269 males, 225 females, and 9 undetermined) in *EB* and 210 dwarf palms (67 males, 49 females, and 94 undetermined) in *PF* (*ca.* 350 palms/ha in both plots). Each year and for each individual palm, we evaluated three types of herbivory: (a) incidence (i.e., yes/no) and intensity (proportion of attacked stems) of moth herbivory. We considered stems as moth-attacked if they showed any visible sign of infection: presence of sawdust, perforated leaves, dried core leaves, twisted or dead stem (Sarto i Monteys and Aguilar 2005); (b) incidence and intensity of goat florivory (proportion of inflorescences partially or fully eaten, out of the total number of inflorescences); and (c) incidence and intensity of goat folivory (proportion of browsed leaves, estimated in 15 randomly selected leaves per individual palm). For goat florivory, only flowering palms were considered in the analyses. Palm traits potentially influencing herbivory, specifically palm size-estimated as total number of stems- and total number of inflorescences, were also recorded.

### Statistical analyses

To study sex differences in the probability and intensity of herbivory on *C.* *humilis*, we fitted several Generalized Linear (Mixed) Models (GLM / GLMM; Zuur et al. 2009) for each type of herbivory (i.e., moth herbivory, goat florivory, and goat folivory) and for each palm population (*EB* and *PF*) separately. For the probability of herbivory, we used as response variable whether or not the palm was attacked (i.e., “1” vs. “0”). For the intensity of herbivory, we used the herbivore-attack rate (as described previously for each type of herbivory), considering only the attacked palms in this case. Both response variables were fitted with a binomial error distribution and a logit link function, or with a quasi-likelihood approach when data was significantly over-dispersed.

Palm sex was the main predictor variable in all models. Palm size was included as a covariate when moth herbivory (Ruiz et al. 2018) or goat folivory (Cornelissen et al. 2008) were the response variables. However, in the case of goat florivory, we used the number of inflorescences (Teixido et al. 2011). In addition, sampling year (2019 and 2020) was specified as a fixed term to test for inter-annual variation, and palm individual as a random effect in the GLMMs for goat herbivory. However, because moth herbivory events accumulate along time and because of the low observed inter-annual variation on moth attack rates, for moth herbivory, only data from one sampling year (2019) were considered. We removed from the analysis palms for which the sex could not be determined. We fitted the models using the R functions *glm* {stats}, *glmer* {lme4}, and *glmmPQL* {MASS}. For each palm sex, we calculated adjusted means and standard errors using the *lsmeans* {lsmeans} function in R software (R Core Team 2023).

### Spatial point pattern analysis (SPPA)

To evaluate whether the occurrence and the intensity of herbivory on *C.* *humilis* plants, as well as their size and number of inflorescences, were influenced by conspecific neighborhood effects and whether these effects differed between female and male dwarf palms, we used recent extensions of spatial point pattern analyses (SPPA, hereafter; Wiegand and Moloney 2014; Fedriani et al. 2015; Velázquez et al. 2016). The point pattern data consist, in our case, of the mapped locations of all dwarf palms within a given observation window, with additional information of particular plant traits called ‘marks’. Palms were characterized by qualitative marks (i.e., female vs. male, attacked *vs.* unattacked) and quantitative marks (i.e., intensity of herbivore attacks, plant size, and number of inflorescences). Specifically, we conducted the following analyses to address our hypotheses (Table 1):

1. To detect spatial patterns in the probability of herbivory, such as spatial aggregation of attacked or unattacked palms, and differences in the density of conspecific neighbors (hypothesis 1), we used analysis of univariate patterns (i.e., male and female palms together) with one qualitative mark (“attacked” vs. “unattacked”). To find out if attacked or unattacked palms were aggregated within all palms, we employed the mark connection functions *p*_11_ or *p*_22_, respectively, which represent the conditional probability of two palms separated by a distance *r* being of the same or different type. We also used the summary function *g*_1,1+2_(*r*)–*g*_2,1+2_(*r*), but normalized in the same way as mark connection functions, to find out if the density of palms at distance *r* of attacked palms is smaller or larger than that of unattacked palms (Wiegand and Moloney 2014).
2. To find out if, and at what distance *r*, the density of conspecific neighbors influences the attack intensity (hypothesis 1), we used analysis of univariate patterns with one quantitative mark. Specifically, we used the density correlation function *C*_m,g_(*r*) (Fedriani et al. 2015; Holík and Janík 2022) to evaluate whether the intensity of attack of palms is positively or negatively correlated with the density of conspecifics at distance *r*. For example, a positive value of *C*_m,g_(*r*) indicates that the attack intensity of palms are positively correlated with the neighborhood density at distance *r*.
3. To assess if the density of neighbors of the same or different sex had an influence on the quantitative marks (i.e., attack intensity, palm size or number of inflorescences; hypotheses 3 and 4), we used analysis of uni- and bivariate qualitatively marked patterns with one quantitative and one qualitative mark (Wiegand and Moloney 2014). This analysis is conceptually more complex because we have two patterns; for example, females are focal pattern 1 and males pattern 2, with *m*_1*i*_ being the quantitative mark of female *i* and *m*_2*j*_ that of male *j*. To evaluate whether the quantitative mark is positively or negatively correlated with the density of female or male palms at a certain neighborhood with radius *r*, we used univariate and bivariate density correlation functions (*C*_m1,g1_(*r*) and *C*_m1,g2_(*r*)). For example, in the case that females are the focal pattern, *C*_m1,g1_(*r*) and *C*_m1,g2_(*r*) are the Pearson correlation coefficients between the quantitative mark *m*_1*i*_ of females *i* and the number of their female (univariate) or male (bivariate) neighbors at distance *r*, respectively (Wiegand and Moloney 2014; Fedriani et al. 2015).

**Table 1:** Summary of the spatial point pattern analyses used to investigate conspecific neighborhood effects on the incidence and intensity of moth herbivory and goat herbivory, and those on key plant traits, such as palm size and number of inflorescences

Study variable	Hypotheses	Summary functions	Spatial effects studied	Null models
Herbivory attack	(1)	Mark connection functions *p*_11_(*r*) and *p*_22_(*r*)	Aggregation of attacked (or unattacked) palms within all palms	Random labeling of mark “attacked” over all palms
		Normalized difference (*g*_1,1+2_(*r*) *–* *g*_2,1+2_(*r*))/*g*_1+2,1+2_(*r*)	Attacked (or unattacked) palms are located in areas of high (or low) palm density	
Intensity of herbivory	(1), (3)	Density correlation function *C*_m,g_(*r*) *C*_m1,g1_(*r*), *C*_m1,g2_(*r*)	Intensity of herbivory is correlated with the neighborhood density of palms	Random marking of the intensity of herbivory over all palms
			Male and female palms differ in their correlation of the intensity of herbivory with the neighborhood density of palms	Random marking of the intensity of herbivory only of the focal sex (pattern 1)
Palm size or number of inflorescences	(4)	Density correlation function *C*_m,g_(*r*) *C*_m1,g1_(*r*), *C*_m1,g2_(*r*)	Male and female palms differ in their correlation of palm size (or number of inflorescences) with the neighborhood density of palms	Random marking of the palm size or number of inflorescences only of the focal sex (pattern 1)

Study variable, hypotheses, summary functions, spatial effects studied and null models used are shown

Then, the observed values of these summary functions were compared to those of 199 simulations of a suitable Monte Carlo null model that represents lack of spatial structure of the marks. In the univariate analyses, we used the random marking null model where the marks are randomly shuffled over all palms, whereas in the bivariate analyses, we kept the quantitative mark of the pattern 2 (e.g., males) fixed and applied the random marking null model to the quantitative mark of pattern 1 (e.g., the intensity of herbivore attack of females). We then conducted a global envelope test which allows us to reject the null hypothesis, with prescribed significance level α, if the empirical summary function is at least once outside the envelopes (Velázquez et al. 2016; Wiegand et al. 2016; Chanthorn et al. 2018). All SPPA were carried out with the free software Programita version 2021 (http://programita.org, Wiegand and Moloney 2014). Given that we collected data during two consecutive years (i.e., temporal replicates) and did not find significant differences between the resulting spatial patterns, we combined the results from the two years in most cases into averaged summary functions (Wiegand and Moloney 2014). As previously explained, we only show the results related to the first sampling year (2019) for moth herbivory and palm size. Moreover, we only used data from the second sampling year (2020) to calculate the mark connection functions (hypothesis 1) for goat florivory as only three palms were unattacked in 2019. More detailed descriptions of the statistical procedures are provided in Additional file 1.

## Results

### Conspecific neighborhood effects on *C. humilis*–herbivore interactions

Differences in moth and goat herbivory were observed between populations and years (see Table S1). In 2019, approximately 10% and 29% of the palms were moth-attacked in *EB* and *PF*, respectively. The intensity of moth herbivory was also nearly three times higher in *PF* compared to *EB.* Moreover, goat florivory was significantly higher in *PF*, with an incidence of 91% compared to 73% in *EB*, and an intensity of 86% compared to 39% in *EB*. Finally, goat folivory (intensity range: 0–100%) did not show any clear spatiotemporal pattern (see Table S1).

**Fig. 1:**
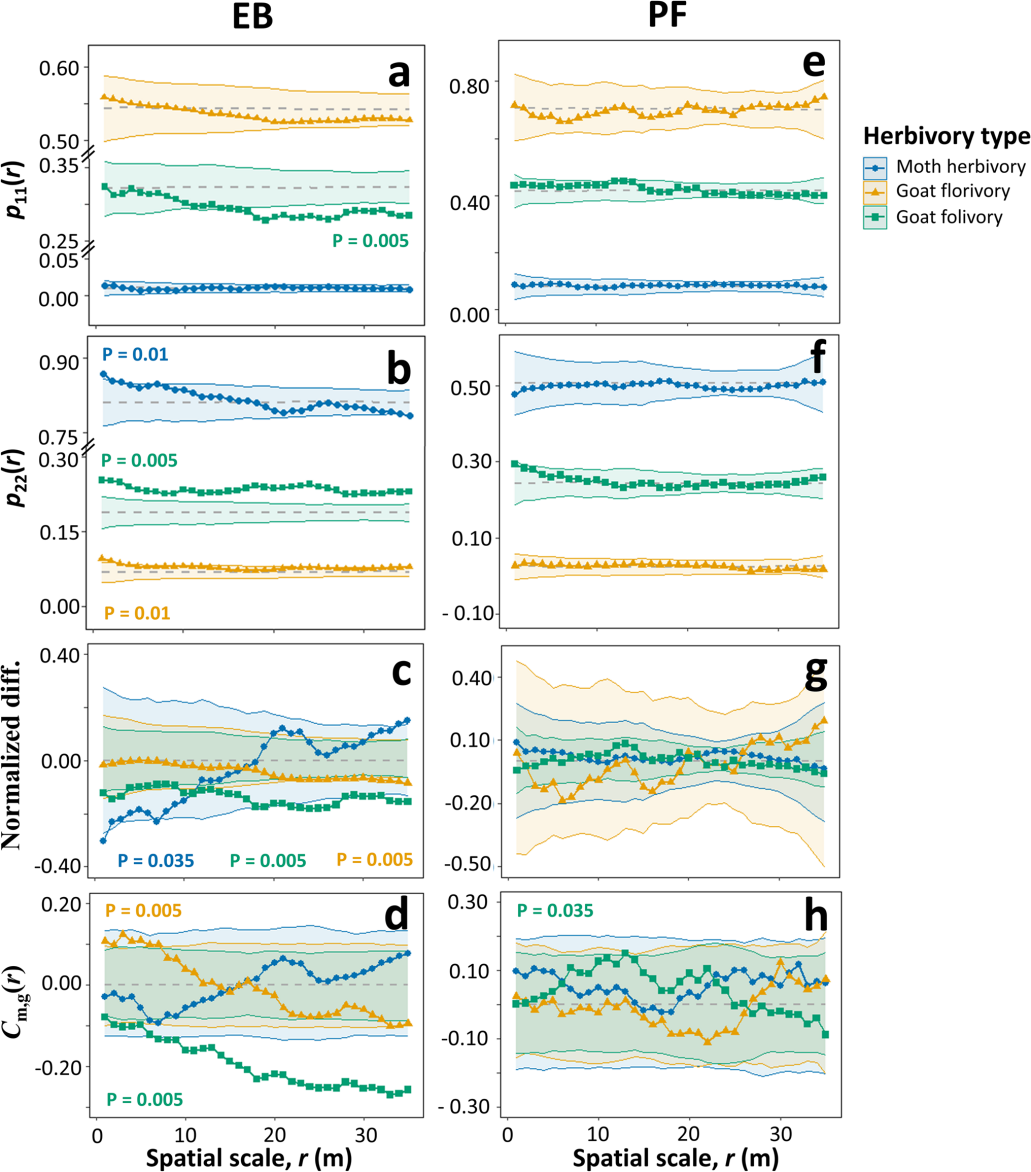
The probability and the intensity of moth herbivory (blue circles), goat florivory (yellow triangles) and goat folivory (green squares) on focal palms related to the distance and density of their conspecific neighbors in *EB* (**a**–**d**) and *PF* (**e**–**h**). The univariate mark connection functions *p*_11_(*r*) or *p*_22_(*r*) investigate if attacked or unattacked palms are aggregated, respectively; the normalized difference function (*g*_1,1+2_(*r*) – *g*_2,1+2_(*r*))/*g*_1+2, 1+2_(*r*) investigates if attacked palms have at distance *r* more palm neighbors (i.e., attacked + unattacked) than unattacked palms; and the univariate density correlation function *C*_m,g_(*r*) estimates the correlation between the herbivore-attack intensity suffered by palms and the number of their neighbors. The gray dashed lines represent the expected functions of the null models, the dotted lines are the functions for the observed data, and the colored shades show the global simulation envelopes for each type of herbivory. *P* values from the GoF test are shown only for significant effects

In *EB*, palms not affected by moth herbivory showed at small distances a significant spatial aggregation within all palms (*p*_22_(*r*), *P* = 0.01; Fig. 1b: blue), whereas attacked palms did not show a significant spatial pattern (*p*_11_(*r*)*,* *P* = 0.73; Fig. 1a: blue). Additionally, unattacked palms had at small distances significantly more conspecific neighbors (i.e., attacked + unattacked palms) than attacked palms ((*g*_1,1+2_(*r*) *–* *g*_2,1+2_(*r*))/*g*_1+2,1+2_(*r*), *P* = 0.03; Fig. 1c: blue), probably because they aggregated at small distances. In contrast, we found no spatial structure in the intensity of moth herbivory when looking at the correlation between the intensity of moth herbivory and the number of neighbors (*P* = 0.055; Fig. 1d: blue; Table 3A, “uni” mf–mf).

Similar to moth herbivory, palms not affected by goat florivory were spatially aggregated at small distances (*p*_22_(*r*), *P* = 0.01; Fig. 1b: yellow), and attacked palms did not exhibit a clear spatial association neither among themselves (*p*_11_(*r*)*,* *P* = 0.26; Fig. 1a: yellow) nor with their neighbors ((*g*_1,1+2_(*r*) *–* *g*_2,1+2_(*r*))/*g*_1+2,1+2_(*r*), *P* = 0.005; Fig. 1c: yellow). In contrast, the intensity of goat florivory increased at high densities of conspecific neighbors up to 8–10 m (*C*_m,g_(*r*), Fig. 1d: yellow, “uni” mf – mf in Table 3A).

Finally, both the probability (Table 2) and intensity of goat folivory (Table 3A, “uni” mf – mf) decreased at high densities of conspecific neighbors. Unattacked palms were aggregated and showed, over all distances investigated, a higher density of conspecific neighbors than attacked palms ((*g*_1,1+2_(*r*) *–* *g*_2,1+2_(*r*))/*g*_1+2,1+2_(*r*), *P* < 0.005; Fig. 1b, c: green). Additionally, the mean fraction of leaves consumed was negatively correlated with the density of neighbors (Rank = 200, *P* = 0.005; Fig. 1d: green).

**Table 2:** Summary of the results of the qualitatively marked point pattern analysis of the *EB* and the *PF* populations

	EB, mark connection functions			PF, mark connection functions		
	*p*_11_	*p*_22_	dif	*p*_11_	*p*_22_	dif
Moth herbivory	000000	+ 00000	− −0000	000000	000000	000000
Goat florivory	000000	+ 00000	000000	000000	000000	000000
Goat folivory	000− − −	+ + + + + +	− − − − − −	000000	000000	000000
Figure	Fig. 1a	Fig. 1b	Fig. 1c	Fig. 1e	Fig. 1f	Fig. 1g

The analysis investigated spatial patterns in moth herbivory, goat florivory and goat folivory. The mark connection functions *p*_*ij*_(*r*) represent the conditional probability that a pair of points separated by a distance *r* are the first of type *i* and the second of type *j*. We analyzed the summary functions *p*_11_(*r*) (i.e., attacked vs. attacked), *p*_22_(*r*) (i.e., unattacked vs. unattacked) and (*g*_1,1+2_(*r*) – *g*_2,1+2_(*r*))/*g*_1+2, 1+2_(*r*) (which compares the number of palms at distance *r* of attacked palms with that of unattacked palms). The six symbols summarize the results of the global envelope test for distance intervals 1–5 m, 6–10 m, …, 25–30 m using the symbols –and + if at a given interval the summary function was at least once below or above the global envelopes, respectively, and 0 otherwise.”dif” stands for the normalized difference function (*g*_1,1+2_(*r*) – *g*_2,1+2_(*r*))/*g*_1+2, 1+2_(*r*)

**Table 3:** Summary of the results of the quantitatively marked point pattern analysis of the *EB* population

EB plot	Non-spatial means			Density correlation functions				
				uni	uni	bi	uni	bi
A) Moth herbivory	mf	f	m	mf – mf	f – f	f – m	m – m	m – f
	0.015	0.006	0.023	000000	000000	000000	000000	000000
Goat florivory	0.37	0.23*	0.51*	+ + 0000	00–000	000000	0000− −	0000−0
Goat folivory	0.14	0.14	0.13	− − − − − −	− − − − − −	0 − − − − −	00− − − −	− − 00 − −
Figure		Fig. 2a	Fig. 2a	Fig. 1d	Fig. 3a	Fig. 3b	Fig. 3c	Fig. 3d
B) Palm size								
	11.6	11.4	11.7		− 00000	000000	− 00000	000000
*N*. inflorescences	19.5	12.0	27.0		−00000	− − − 000	− − − − 00	− − − − 00
Figure					Fig. S1	Fig. S1	Fig. S1	Fig. S1

The analysis investigated spatial patterns in the intensity of moth herbivory, goat florivory, and goat folivory (A), and in palm size and number of inflorescences (B). The non-spatial means refer to the observed mean values for herbivory, palm size, and number of inflorescences. The asterisks indicate statistically significant differences between palm sexes, based on the GLMs and the GLMMs conducted. The density correlation functions reveal, for example, if the mean attack intensity of palms of type 1 is negatively (−), positively ( +), or not (0) correlated with the number of palms of type 2 at distance *r.* Palms of type 1 and 2 can be “mf” (males and females), “f” (females), or “m” (males). The f – m analysis, for example, estimates the correlation coefficient between the mean attack intensity of female palms (type 1) and the number of male palms (type 2) at distance *r*. Symbol conventions as in Table 1

In summary, in the *EB* palm population, invasive moths attacked isolated palms more frequently, whereas goats consumed inflorescences more intensely but leaves less intensely when palms were aggregated. Remarkably, the *PF* palm population exhibited little spatial structure in the pattern of attack by both herbivores (Tables 2 and 4A “uni” mf – mf; Fig. 1e–h). See Figs. S1 and S2 for results in separate panels depending on the herbivory type.

**Table 4:** Same as Table 3, but for the *PF* population

PF plot	Non-spatial means			Density correlation functions				
				uni	uni	bi	uni	bi
A)	mf	f	m	mf – mf	f – f	f – m	m – m	m – f
Moth herbivory	0.13	0.098	0.15	000000	000000	000000	000000	000000
Goat florivory	0.83	0.73	0.90	000000	000000	000000	000000	000000
Goat folivory	0.14	0.14	0.14	00 + 000	000000	000000	000000	0 + + 000
Figure		Fig. 2b	Fig. 2b	Fig. 1h	Fig. 3e	Fig. 3f	Fig. 3g	Fig. 3h
B) Palm size								
	5.08	4.78	5.39		000000	000000	000000	000000
*N*. inflorescences	2.89	2.98	2.80		000000	000000	0000–0	000000
Figure					Fig. S1	Fig. S1	Fig. S1	Fig. S1

### Sex differences in the probability and intensity of moth and goat herbivory

**Fig. 2:**
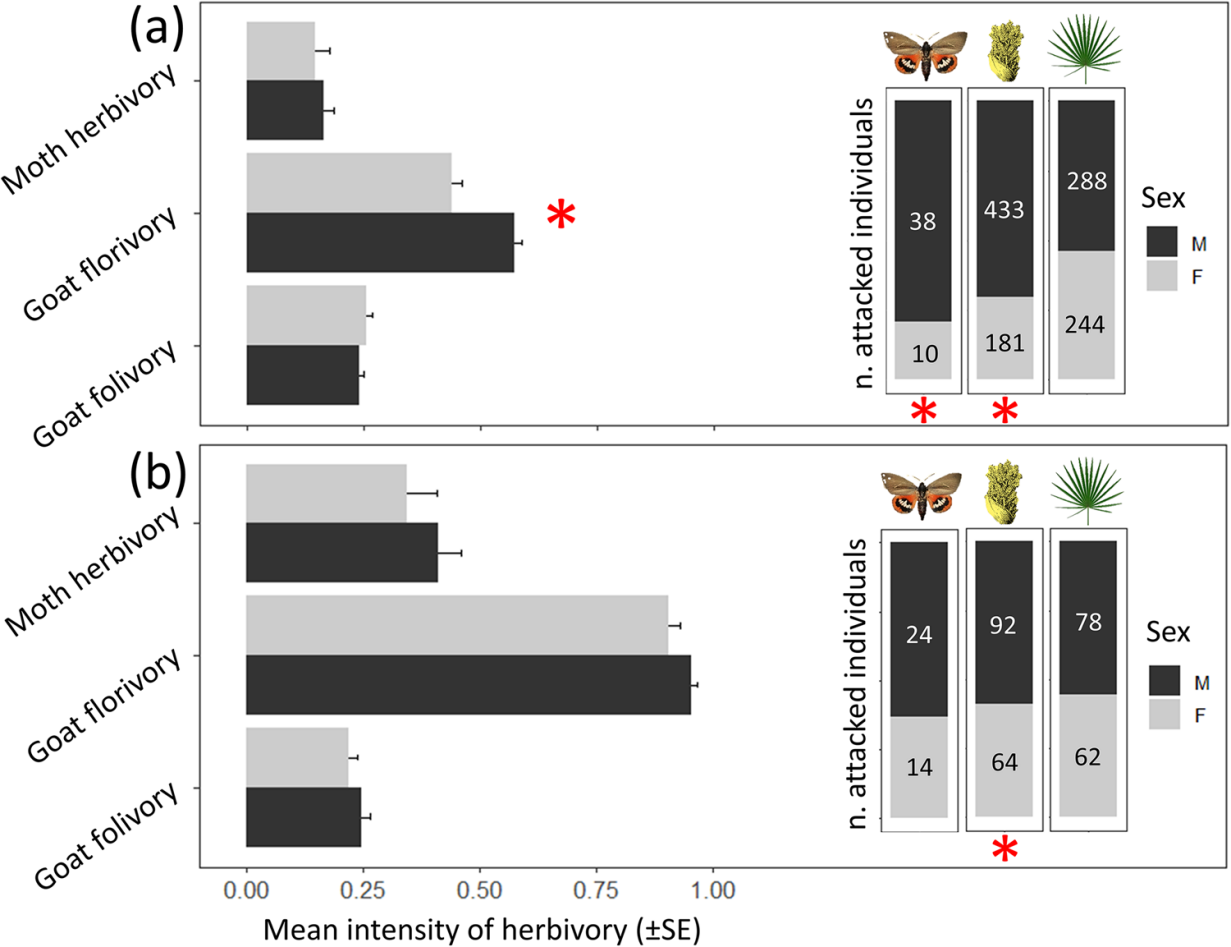
Differences between palm sexes (female, F, in gray; male, M, in black) in the incidence and the mean intensity of moth and goat herbivory in **a** *EB* and **b** *PF*. The insets on the right show the observed number of individuals affected by moth herbivory, goat florivory and goat folivory depending on the palm sex. On the left, the observed mean intensities (± standard error, SE) of moth herbivory, goat florivory and goat folivory of the herbivore-attacked palms from the insets. Red asterisks indicate statistically significant differences (*P* < 0.05) between sexes based on the GLMs and GLMMs conducted in the study. Note that for moth herbivory we only show data from 2019, while for goat florivory and folivory we sum palms from both sampling years (2019 + 2020)

Both populations exhibited a male-biased sex ratio (1:1.2 and 1:1.4 in *EB* and *PF*, respectively). As expected, female and male palms in both populations suffered markedly different probabilities and intensities of herbivory (Fig. 2). In *EB*, males were three times more likely to be moth-attacked (*χ*^2^ = 12.90, *df* = 1, *P* = 0.003) and 1.2 times more likely to experience goat florivory (*χ*^2^ = 45.80, *df* = 1, *P* < 0.001) compared to females (inset Fig. 2A). The intensity of goat florivory was also significantly higher in males (*χ*^2^ = 45.81, *df* = 1, *P* < 0.001), but no differences were found for the intensity of moth herbivory (*χ*^2^ = 0.07, *df* = 1, *P* = 0.80; Fig. 2A). Furthermore, large palms were more prone to experience moth herbivory (*χ*^2^ = 8.17, *df* = 1, *P* = 0.004) and goat folivory (*χ*^2^ = 28.82, *df* = 1, *P* < 0.001). Similarly, the probability of goat florivory increased with the number of inflorescences (*χ*^2^ = 57.87, *df* = 1, *P* < 0.001). However, the intensities of moth herbivory and goat florivory were negatively correlated with palm size (*χ*^2^ = 17.00, *df* = 1, *P* < 0.001) and the number of inflorescences (*χ*^2^ = 90.52, *df* = 1, *P* < 0.001), respectively. In *PF*, males were also more frequently attacked although differences were weaker than in *EB*. Compared to females, males had a 1.05-fold higher probability of goat florivory (*χ*^2^ = 5.93, *df* = 1, *P* = 0.01; inset Fig. 2B), but there was no difference between sexes in the intensity (*χ*^2^ = 1.16, *df* = 1, *P* = 0.28; Fig. 2B). Furthermore, moth herbivory did not significantly differ between sexes in this population (*χ*^2^ ≤ 0.86, *df* = 1, *P* ≥ 0.35), nor did goat folivory in both populations (*χ*^2^ ≤ 0.74, *df* = 1, *P* ≥ 0.39; Fig. 2). Palm size and the number of inflorescences influenced herbivory similarly to *EB*. However, the intensity of goat florivory increased with the abundance of inflorescences (*χ*^2^ = 5.78, *df* = 1, *P* = 0.02).

### Sex-specific neighborhood effects on moth and goat herbivory

**Fig. 3:**
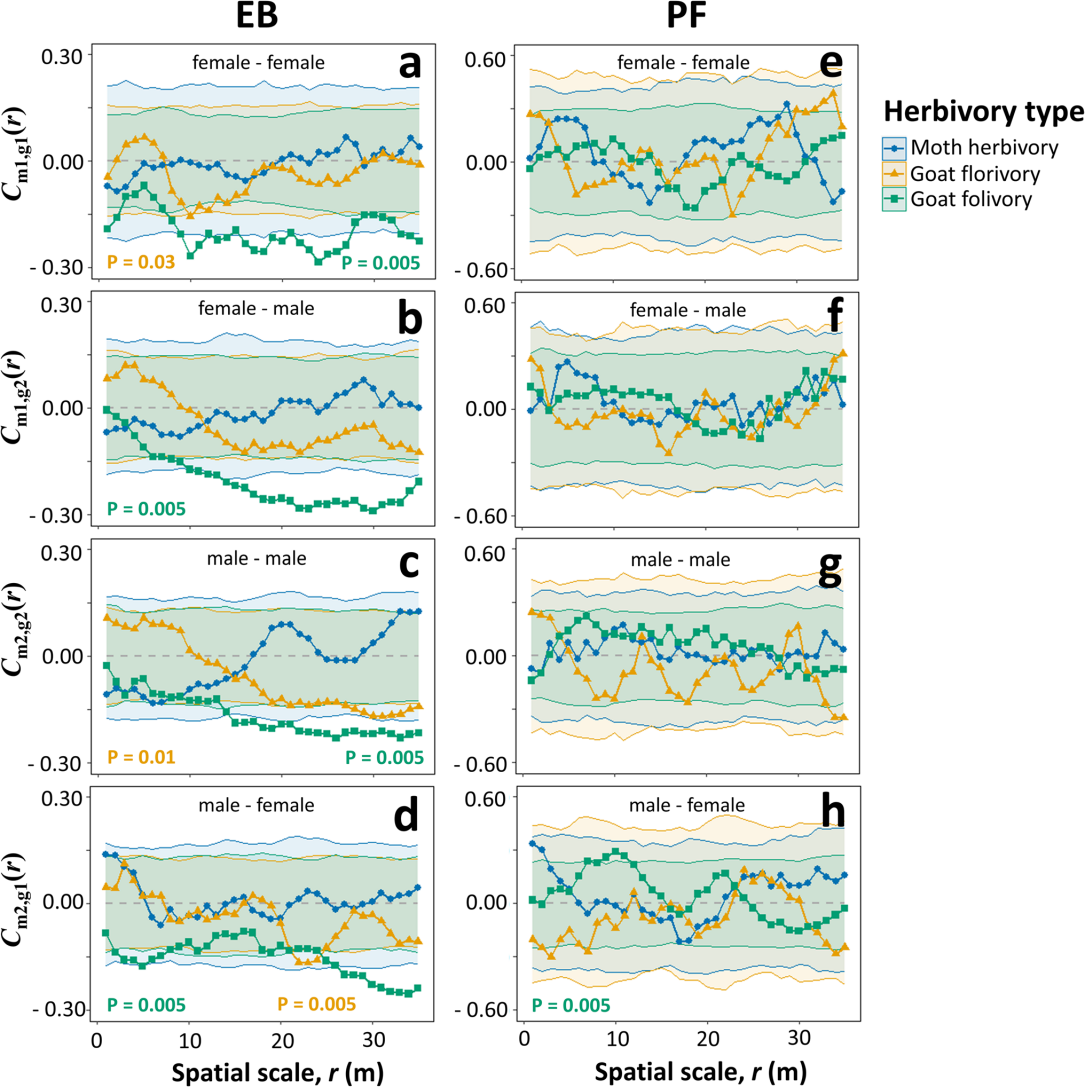
The correlation between the intensity of moth herbivory (blue circles), goat florivory (yellow triangles) and goat folivory (green squares) on focal palms and the density of their conspecific neighbors at distance *r* depending on the palm sex (female *vs.* male) in *EB* (**a**–**d**) and *PF* (**e**–**h**). The univariate density correlation functions *C*_m1,g1_(*r*) and *C*_m2,g2_(*r*) estimate the correlation between the herbivore-attack intensity of female or male palms, respectively, and the number of neighbors of the same sex at distance *r*. The bivariate density correlation functions *C*_m1,g2_(*r*) and *C*_m2,g1_(*r*) estimate the correlation between the herbivore-attack intensity of female or male palms, respectively, and the number of neighbors of the opposite sex at distance *r*. The gray dashed lines represent the expected functions of the null models, the dotted lines are the functions for the observed data, and the colored shades show the global simulation envelopes for each type of herbivory. *P* values from the GoF test are shown only for significant effects

The effect of the distance and the density of conspecific neighbors on herbivory varied between palm sexes in both studied populations (see Tables 3 and 4). In *EB*, female palms experienced lower rates of goat florivory when they had more female neighbors at distances of 10–15 m (*P* = 0.03; Fig. 3a: yellow; Table 3A “uni” f – f), while males tended to experience higher rates when they had more conspecific neighbors, regardless of sex, within 10 m (*P* ≤ 0.10; Fig. 3c, d: yellow; Table 3A “uni” m – m, “bi” m – f). However, there were no sex differences in moth herbivory and goat folivory (Table 3A). For example, both female and male palms showed lower rates of goat folivory when they were surrounded by more conspecific neighbors, regardless of sex, nearby (*P* = 0.005; Fig. 3a–d: green). In *PF*, dwarf palms did not show any significant neighborhood effect on herbivory (Table 4A; *P* ≥ 0.10), except for goat folivory. In male palms, the fraction of leaves consumed by goats increased with the density of female neighbors at distances of 8–12 m (*P* = 0.005; Fig. 3h: green). See Figs. S3 and S4 for results in separate panels depending on the herbivory type.

### The influence of conspecific neighborhood and plant sex on palm size and inflorescence abundance

Palms in EB were twice as large and produced twice as many inflorescences as palms in *PF* (Table S1). Palm size did not differ between sexes in either of the two populations, but in *EB* male palms had twice as many inflorescences on average (27.0 ± 1.3, *n* = 351) as females (12.0 ± 0.7, *n* = 489).

In *EB*, both female and male palms were smaller when they had neighbors of the same sex at short distances, up to 5 m (*P* = 0.005 and *P* = 0.04, respectively; Fig. S5a, c: green; Table 3B, “uni” f-f and m-m), although this effect was stronger in females. In contrast, palm size did not show any significant spatial pattern between sexes in *PF* (*P* ≥ 0.33; Fig. S5e–h; Table 4B). With regard to the number of inflorescences, both female and male palms produced fewer inflorescences at high densities of conspecific neighbors up to 15–20 m of distance, regardless of the neighbor’s sex, in *EB* (*P* = 0.005; Fig. S5a–d: yellow; Table 3B). However, we did not find clear patterns for the number of inflorescences in *PF* (*P* ≥ 0.01; Fig. S5e–h: yellow; Table 4B).

## Discussion

Our spatial analyses revealed that herbivory by the invasive moth *P.* *archon* and the feral goat *C.* *hircus* on the Mediterranean palm *C.* *humilis* is influenced in complex ways by the density of neighbors at particular distances, plant sex, and other plant traits, such as size and number of inflorescences. For example, isolated palms (i.e., with low densities of conspecific neighbors) were more prone to be moth-attacked and experience more goat folivory, while aggregated palms (i.e., with high densities of conspecific neighbors) showed stronger goat florivory. Importantly, these density-dependent effects changed across spatial scales and between palm sexes. Female and male palms exhibited opposite patterns in goat florivory, a result explained by a goat preference for male inflorescences and the observed between-sex differences in palm size and inflorescence abundance. Our results thus highlight the role of plant density and intraspecific plant traits, such as plant sex, in shaping the spatial structure of plant–herbivore interactions.

### Distance- and density-dependent processes of *C. humilis* in its interaction with two contrasting herbivores

The different neighborhood effects observed for each type of herbivory could be attributed to several mechanisms. The preference of *P.* *archon* for isolated palms could be explained by the larger size of these individuals as well as the territorial behavior exerted by male moths, which usually remain in reduced areas to attract females (Sarto i Monteys and Aguilar 2005; Liégeois et al. 2016; Quero et al. 2017). It is also possible, as hypothesized by Ruiz et al. (2018), that female moths avoid attacked palms to reduce intraspecific competition of larvae (Sarto i Monteys and Aguilar 2005). An interesting finding was that the relationship between goat herbivory and the neighborhood density of palms changed depending on the plant resource consumed (i.e., inflorescences vs. leaves). We attribute these differences to the feeding preferences and the contrasting temporal availabilities of inflorescences and leaves. On the one hand, inflorescences are a very ephemeral resource (only available a few weeks a year), which is likely much more nutritious and, thus, preferred over palm leaves. Therefore, a resource concentration effect (Root 1973) may emerge that attracts more herbivores the higher the density of palms. For instance, Knegt et al. (2007) found that goats concentrate their foraging effort in areas with high reward, in our case the palm inflorescences. On the other hand, leaves are a superabundant and permanently available resource, leading to a resource dilution effect (Otway et al. 2005). Goats are likely to avoid palms with high densities of neighbors because these palms are generally smaller than isolated palms and offer fewer leaves, or because goats cannot easily exploit dense palm groups.

The spatial scaling of the neighborhood effects also changed depending on the herbivory type. While moth herbivory and goat florivory on a focal palm were influenced by conspecific neighbors at short distances, up to 10 m, probability and intensity of goat folivory decreased at distances up to 35 m. Although neighborhood effects at short distances are more common (e.g., Palmer et al. 2003; Holík et al. 2021), large-scale effects have also been reported. Deer browsing on *Abies* *balsamea* seedlings negatively correlated with the nutritional quality of conspecific seedlings at distances up to 50 m (Champagne et al. 2018). In a lowland alluvial forest, the probability of bark stripping by deer on hetero-specific trees decreased up to a distance of 25 m from *Carpinus* trees (Holík and Janík 2022). Such large-scale neighborhood effects can be explained by the foraging behavior of herbivores (Fryxell et al. 2008) or by spatial heterogeneity in the pattern of plants (Wiegand and Moloney 2014). In our study case, large-scale neighborhood effects on goat folivory were detected even when applying local marking null models (e.g., Holík and Janík 2022), which reduces the influence of spatial heterogeneity. Therefore, differences in the spatial scaling between goat florivory and goat folivory indicate that goat-feeding behavior is dependent on the abundance and type of food resource (inflorescences *vs.* leaves).

The lack of neighborhood effects on herbivory in one of our study plots (the *PF* plot) could result from extreme levels of herbivory. Intense herbivore pressure is demonstrated to weaken or even cancel spatial patterns as herbivore selectiveness decreases (Ohgushi 2005; Baraza et al. 2006; Wang et al. 2020). In this sense, severe herbivory by goats may have led to a simplified spatial structure of the palm population (i.e., low degree of clustering and decreased recruitment rates; Muñoz-Gallego et al. 2023) and a decreased palm size.

Although *C.* *humilis* was the predominant plant species in both study plots and other species showed very low abundances, the hetero-specific neighborhood may also influence the observed patterns (Kim and Underwood 2015; Downey et al. 2018; Holík and Janík 2022). Future investigations should therefore also consider how other neighboring plant species affect moth and goat herbivory on dwarf palms.

### Plant sex effects in the dwarf palm-herbivore interactions

Sex-specific effects in biotic interactions (e.g.,Vega-Frutis et al. 2013; Moritz et al. 2016; Zhang and He 2017; Mosseler et al. 2020; Martín-Forés et al. 2022), and specifically, in plant–herbivore interactions (Cornelissen and Stiling 2005; Avila-Sakar and Romanow 2012; Moritz et al. 2018; Liu et al. 2021) have been widely described in the literature. Although Ruiz et al. (2018) found no evidence that palm sex influenced moth herbivory, in our study, male palms were more likely to be attacked by *P.* *archon* and were more likely to be affected by goat florivory than female palms. This result supports the male-biased herbivory hypothesis (Cornelissen and Stiling 2005; Cepeda-Cornejo and Dirzo 2010).

Concerning moth herbivory, female palms may be less suitable than male palms for oviposition and larval development, probably due to morphological and/or chemical mechanisms. The study by Tsuji and Sota (2010), for instance, found that female flowers of the sexually polymorphic shrub *Eurya* *japonica* prevent larval development of the moth *Chloroclystis* *excise* owing to high concentrations of phenolics and tannins within the calyces. In two other palm species, male individuals of *Phoenix* *canariensis* *and* *P.* *dactylifera* were more often infested by *Rhynchophorus* *ferrugineus* than females because higher amounts of chemical attractants and a less fibrous crown make them more suitable for oviposition (Rochat et al. 2017; Al Ansi et al. 2020). Furthermore, Jácome-Flores et al. (2018) found that larval development of the dwarf palm pollinator *Derelomus* *chamaeropis* happens mostly in male palms; thus, female palms might be also better defended against herbivory by the invasive moth *P.* *archon*.

The feeding preference of goats for male over female palm inflorescences in both palm populations could be explained by three nonexclusive hypotheses: (a) Male palms offer more abundant rewards, as described for other dioecious plants (McCall and Irwin 2006; Barrett and Hough 2012). Besides male palms had twice as many inflorescences as females in one of our study populations, male inflorescences usually produce more flowers per inflorescence than do female inflorescences (Herrera 1989b). (b) Male inflorescences and flowers often offer higher nutritional qualities as well as higher rewards, such as rich nitrogen content in pollen, compared to females (Cornelissen and Stiling 2005; McCall and Irwin 2006; Barrett and Hough 2012; Ndem-Galbert et al. 2021). (c) Male inflorescences may be less defended than female inflorescences. It is well known that reproductive tissues of females have secondary compounds and additional defenses against herbivory to protect the flowers and developing fruits to ensure successful reproduction (Cornelissen and Stiling 2005; Cepeda-Cornejo and Dirzo 2010; Tsuji and Sota 2010). In fact, female flowers and developing fruits of *C.* *humilis* secrete a protective resin with high amounts of amino acids that may prevent herbivory and fruit predation (Herrera 1989a; Fedriani and Delibes 2011).

### Plant sex as a driver of neighborhood effects on herbivory

Sexual dimorphism in dioecious species often results in trait differences between females and males, which could lead to different levels of herbivory and plant neighborhood effects. Our research revealed that palm sexes exhibited opposite neighborhood effects. For example, the intensity of goat florivory on female palms decreased at high densities of female neighbors at a distance of 10–15 m, while it tended to increase on male palms at high densities of neighbors, regardless of sex. Thus, in terms of reproductive success (Muñoz-Gallego et al. 2022), palm aggregation has a positive effect for females as goat florivory decreased, but a negative effect for males as goat florivory increased. Several factors may contribute to these patterns: (1) goats prefer male over female inflorescences, (2) female palms produce fewer inflorescences compared to male palms, and (3) the negative relationship between palm size and the number of conspecific neighbors of the same sex is stronger in females. Although this pattern was only observed in one of our two study populations and its magnitude was marginal, our findings highlight that sex-specific plant neighborhood effects could be masked when focusing only on general patterns within a plant population. This suggests that other dioecious species with striking differences between female and male individuals, such as those found in the genus *Leucadendron* (Proteaceae; see Fig. 1 in Barrett and Hough 2012), may also exhibit neighborhood effects on herbivory driven by plant sex. Consequently, we encourage for further research on the spatial ecology of herbivory driven by sex differences.

## Conclusions

Our study provides new insights into how density and traits of conspecific neighbors modulate the probability and intensity of herbivory on a focal plant. Using techniques of spatial point pattern analysis that allow exploration over a range of neighborhood distances, we show that neighborhood effects can vary across scales, herbivory types, and more interestingly, between plant sexes in the Mediterranean palm *C.* *humilis.* Our results suggest that plant sex is a significant driver of neighborhood effects on dioecious species, in which sexual dimorphism has been demonstrated to influence herbivore preference (Cornelissen and Stiling 2005). However, to obtain a more general understanding of the role of plant sex and other relevant traits in shaping plant–herbivore interactions, future research should be conducted on other herbivore and dioecious plant species, considering hetero-specific neighborhood effects across a range of scales (e.g., Holík and Janík 2022).

### Supplementary Information

Below is the link to the electronic supplementary material.

Supplementary file1 (PDF 1197 KB)

## Data Availability

Data are available on FigShare at 10.6084/m9.figshare.24212508.v1.
