# Supplementary material for: Sex-driven neighborhood effects on herbivory in the dioecious Mediterranean palm Chamaerops humilis L
Source: Oecologia. 2023 Oct 5;203(1-2):151–65. doi: 10.1007/s00442-023-05457-z (PMC10615982; doi:10.1007/s00442-023-05457-z)

Supplementary Material

Sex-driven neighborhood effects on herbivory in the dioecious Mediterranean palm *Chamaerops humilis* L.

Raquel Muñoz-Gallego[1]*; Thorsten Wiegand[2,3]; Anna Traveset[1] & Jose M. Fedriani[4,5]

[1] Global Change Research Group, Mediterranean Institute of Advanced Studies (IMEDEA, CSIC-UIB), (C/ Miquel Marquès, 21, 07190, Esporles, Balearic Islands, Spain)

[2] Department of Ecological Modelling, Helmholtz Centre for Environmental Research GmbH – UFZ. Leipzig (Germany).

[3] German Centre for Integrative Biodiversity Research (iDiv) Halle-Jena-Leipzig, Leipzig, Germany.

[4] Desertification Research Centre (CIDE, CSIC), (Crta. Moncada-Náquera, Km 4.5, 46113, Moncada, Valencia, Spain).

[5] Doñana Biological Station (EBD, CSIC), (C/Americo Vespucio s/n, 41092 Seville, Spain).

*First corresponding author: Raquel Muñoz Gallego, rmunoz@imedea.uib-csic.es

*Second corresponding author: Jose M. Fedriani, fedriani@csic.es

Table of contents

1. Definition of the test statistics
2. Supplementary tables
3. Supplementary figures

1. *Definition of the test statistics (Wiegand and Moloney, 2014; Fedriani et al., 2015):*

- **Mark connection functions $p_{ij}(r)$** are the adapted summary functions of quantitatively marked patterns. In this data structure each point carries a point, either of type 1 or of type 2 (e.g., attacked vs. unattacked). The interest in the analysis of qualitatively marked patterns is e.g., to find out how strongly attacked palms are aggregated within the joined pattern of all palms. This requires to remove the confounding spatial effect of the unmarked pattern (i.e., formed by all palms of type 1 and type 2), which is contained in the corresponding pair correlation functions $g_{ij}(r)$. Mark connection functions accomplish this by dividing $g_{ij}(r)$ by the pair correlation function $g_{1+2,1+2}(r)$ of the unmarked pattern:

$$p_{ij}(r) = p_i p_j \frac{g_{ij}(r)}{g_{1+2,1+2}(r)}$$

  where the $g_{ij}(r)$ are partial or bivariate pair correlation function that quantifies the relative density of type *j* plants around type *i* plants (where *i* and *j* can have values of 1 or 2), $g_{1+2,1+2}(r)$ is the pair correlation function of the unmarked pattern, and p_i is the proportion of type *i* plants among all plants.

  Specifically, the **mark connection function $p_{11}(r)$** measures the conditional probability that both of two plants separated by distance *r* have the qualitative mark 1. If plants of type 1 are a random sample of all plants, we expect $p_{11}(r) = p_1p_1$. If they are aggregated within all plants, we find $p_{11}(r) > p_1p_1$. If they are isolated within all plants, we find $p_{11}(r) < p_1p_1$. The **mark connection function $p_{22}(r)$** measures the conditional probability that both of two plants separated by distance *r* have the qualitative mark 2. To calculate it, we use the same equation as for $p_{11}(r)$.

- **Test statistic $g_{1,1+2}(r)$ - $g_{2,1+2}(r)$:** It indicates whether plants of type 1 are preferentially located in areas of overall high plant density (i.e. clusters). This test statistic compares the density of plants (i.e. 1+2) around plants of type 1 [$g_{1,1+2}(r)$] with the density of plants (i.e. 1+2) around

plants of type 2 [$g_{2,1+2}(r)$]. The expected value of this test statistics is zero under random labelling, but if plants of type 1 occur disproportionately within plant clusters we expect $g_{1,1+2}(r) > g_{2,1+2}(r)$. Given that this summary statistic still conserves the signal of the underlying unmarked pattern, we also normalize it by the pair correlation function of the unmarked pattern [$(g_{1,1+2}(r) - g_{2,1+2}(r))/g_{1+2,1+2}(r)$]. In this way, the difference is expressed as fraction of the average neighborhood density of palms at the given distance r. This allows for a more direct interpretation of the strength of the density effect because the normalized difference factors out the effect of underlying spatial pattern of all palms.

- **Density correlation function $C_{m,g}(r)$:** It estimates the classical Pearson correlation coefficient between the quantitative mark m_i of a plant and the number of neighbors at distance r [$=\lambda g_i(r)$]. Thus, the density correlation function is based on the following test function:

$$t(r,m_i,g_i) = [m_i - \mu][(\lambda g_i(r) - \lambda g(r)]$$

where m_i is the quantitative mark of the focal plant i, μ is the mean quantitative mark of the population, λ the overall density of plants in the study area, $\lambda g_i(r)$ the density of neighbors around the focal plant i at distance r, and $\lambda g(r)$ the mean density of neighbors at distance r for all trees. The $g_i(r)$ is basically a 'local' g-function, and the average over all $g_i(r)$'s yields the well-known pair-correlation function $g(r)$. Estimation of $g_i(r)$ requires an edge correction factor w_i if the neighborhood around plant i is not fully within the observation window. The density correlation function $C_{m,g}(r)$ is normalized by the product of the standard deviations $\sigma_m\sigma_g$ of the marks m_i and the individual g-functions $g_i(r)$, respectively. 'C' stands for correlation, 'm' for the first mark m_i and 'g' for the second mark $g_i(r)$.

2. *Supplementary tables:*

Table S1. Descriptive variables of herbivory: incidence of moth herbivory, goat florivory and goat folivory (%), mean intensity of moth herbivory (i.e. proportion of attacked stems/palm), mean intensity of goat florivory (i.e. the proportion of eaten inflorescences/palm), mean intensity of goat folivory (i.e. the proportion of browsed leaves over 15 leaves randomly selected/palm), mean inflorescence production (i.e. the number of inflorescences/stem/palm), mean palm size (i.e. total number of stems/palm) and sex ratio for both palm populations (*EB* and *PF*) and sampling years (2019 and 2020).

	**Ermita de Betlem (*EB*)**		**Platja de Formentor (*PF*)**	
	**2019**	**2020**	**2019**	**2020**
**Moth-attacked individuals (%)**	10.12 (503)	15.66 (466)	29.05 (210)	40.00 (205)
**Individuals affected by goat florivory**	75.10 (478)	71.65 (381)	97.78 (135)	84.11 (107)
**Individuals affected by goat folivory**	60.32 (503)	52.15 (466)	84.76 (210)	31.22 (205)
**Intensity of moth herbivory (mean ± SE)**	0.015 ± 0.003 (503)	0.024 ± 0.003 (466)	0.106 ± 0.014 (210)	0.144 ± 0.016 (205)
**Intensity of goat florivory**	0.39 ± 0.01 (478)	0.39 ± 0.02 (381)	0.94 ± 0.02 (135)	0.79 ± 0.04 (107)
**Intensity of goat folivory**	0.16 ± 0.01 (503)	0.12 ± 0.01 (466)	0.24 ± 0.01 (210)	0.05 ± 0.01 (205)
**Inflorescence production**	1.91 ± 0.07 (478)	1.82 ± 0.09 (381)	0.80 ± 0.05 (135)	0.80 ± 0.09 (107)
**Palm size**	11.71 ± 0.39 (503)	10.58 ± 0.37 (466)	5.22 ± 0.16 (210)	4.64 ± 0.15 (205)
**Females/males/undetermined**	225/269/9	207/250/9	49/67/94	49/67/89
**Sex ratio (female:male)**	1:1.2	1:1.2	1:1.4	1:1.4

**Note that sample size for both the incidence and intensity of goat florivory, and for inflorescence production is the total number of flowering palms.*

3. *Supplementary figures:*

Figure S1. The probability and intensity of moth herbivory (blue), goat florivory (yellow) and goat folivory (green) on focal palms related to the distance and density of their conspecific neighbors in the *EB* plot. The normalized difference function $(g_{1,1+2}(r) - g_{2,1+2}(r))/g_{1+2,\,1+2}(r)$ investigates if attacked palms have at distance r more palm neighbors (i.e., attacked + unattacked) than unattacked palms; and the univariate density correlation function $C_{m,g}(r)$ estimates the correlation between the herbivore-attack intensity suffered by palms and the number of their neighbors. The grey dashed lines represent the expected functions of the null models, the dotted lines are the functions for the observed data, and the colored shades show the global simulation envelopes for each type of herbivory. *P* values from the GoF test are shown only for significant effects. Note that Figures S1a-c and Figures S1d-f refer to Figure 1c and Figure 1d from the main document, respectively.

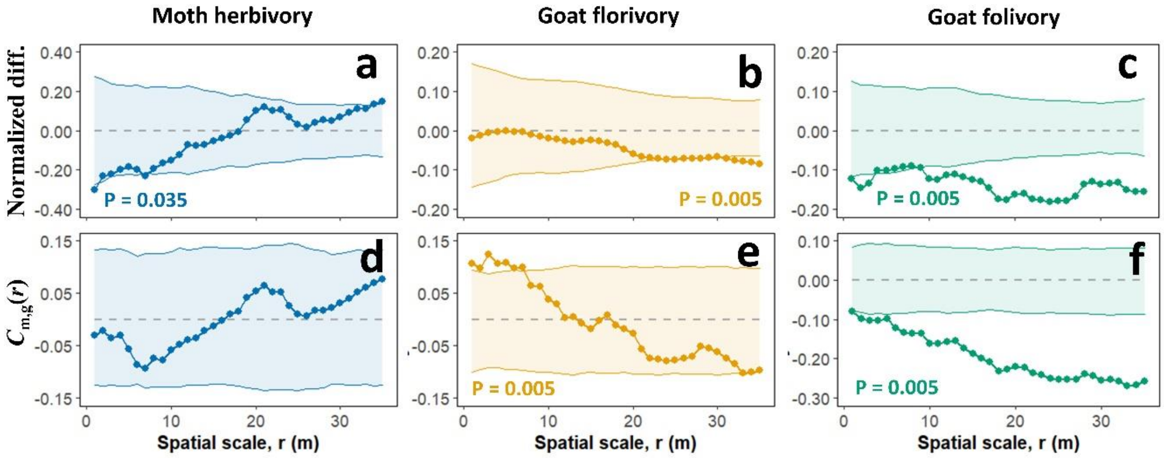

Figure S2. The probability and intensity of moth herbivory (blue), goat florivory (yellow) and goat folivory (green) on focal palms related to the distance and density of their conspecific neighbors in the *PF* plot. The normalized difference function $(g_{1,1+2}(r) - g_{2,1+2}(r))/g_{1+2,\,1+2}(r)$ investigates if attacked palms have at distance r more palm neighbors (i.e., attacked + unattacked) than unattacked palms; and the univariate density correlation function $C_{m,g}(r)$ estimates the correlation between the herbivore-attack intensity suffered by palms and the number of their neighbors. The grey dashed lines represent the expected functions of the null models, the dotted lines are the functions for the observed data, and the colored shades show the global simulation envelopes for each type of herbivory. *P* values from the GoF test are shown only for significant effects. Note that Figures S2a-c and Figures S2d-f refer to Figure 1g and Figure 1h from the main document, respectively.

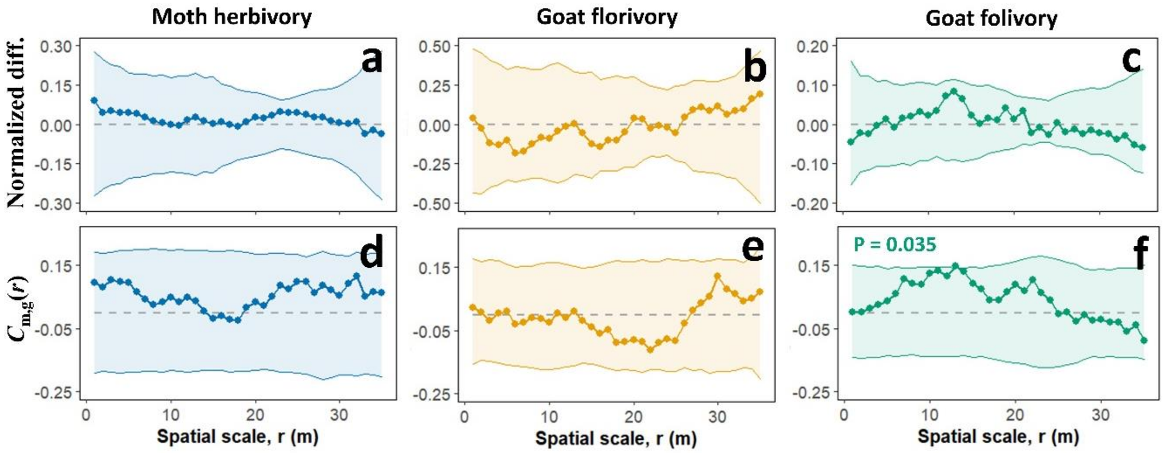

Figure S3. The correlation between the intensity of moth herbivory (blue), goat florivory (yellow) and goat folivory (green) on focal palms and the density of their conspecific neighbors at distance r depending on the palm sex (female *vs.* male) in the *EB* plot. The univariate density correlation functions $C_{m1,g1}(r)$ and $C_{m2,g2}(r)$ estimate the correlation between the herbivore-attack intensity of female or male palms, respectively, and the number of neighbors of the same sex at distance r. The bivariate density correlation functions $C_{m1,g2}(r)$ and $C_{m2,g1}(r)$ estimate the correlation between the herbivore-attack intensity of female or male palms, respectively, and the number of neighbors of the opposite sex at distance r. The grey dashed lines represent the expected functions of the null models, the dotted lines are the functions for the observed data, and the colored shades show the global simulation envelopes for each type of herbivory. P values from the GoF test are shown only for significant effects. Note that Figures S3a-c refer to Figure 3a (female-female), Figures S3d-f to Figure 3b (female-male), Figures S3g-i to Figure 3c (male-male), and Figures j-l to Figure 3d (male-female) from the main document.

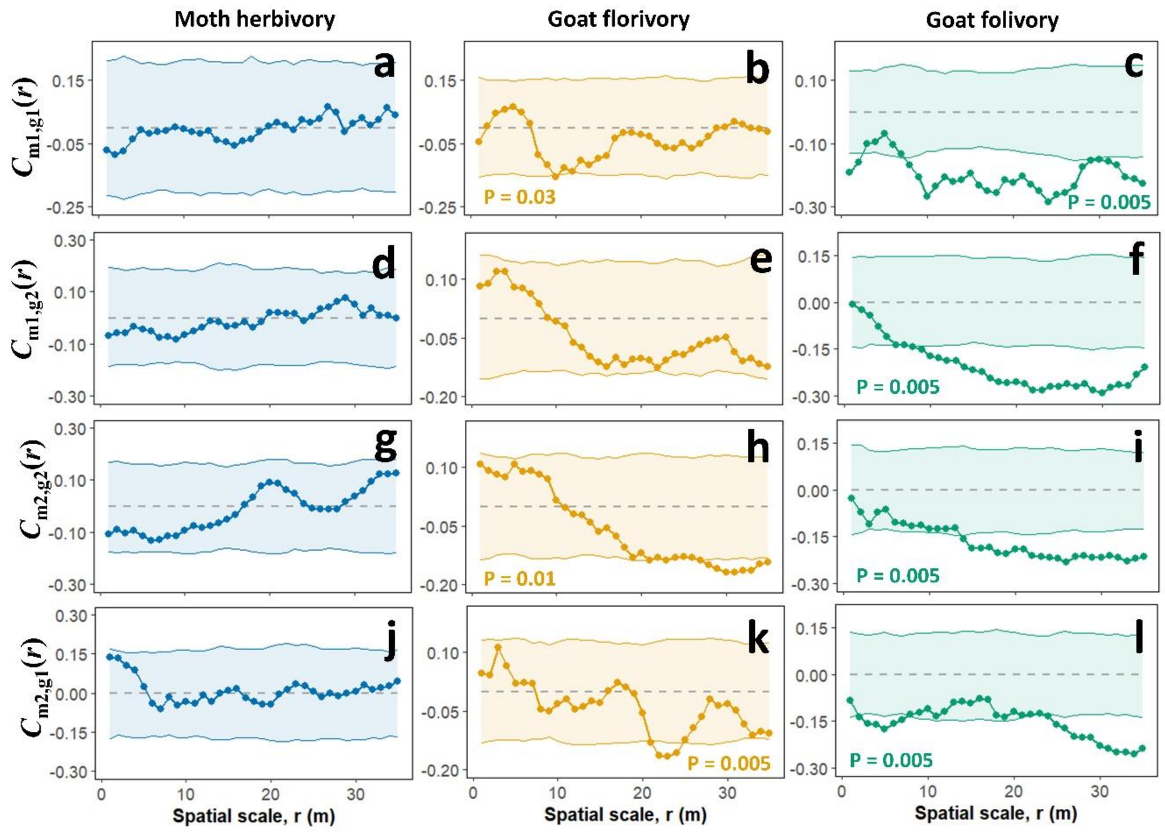

Figure S4. The correlation between the intensity of moth herbivory (blue), goat florivory (yellow) and goat folivory (green) on focal palms and the density of their conspecific neighbors at distance *r* depending on the palm sex (female *vs.* male) in the *PF* plot. The univariate density correlation functions $C_{m1,g1}(r)$ and $C_{m2,g2}(r)$ estimate the correlation between the herbivore-attack intensity of female or male palms, respectively, and the number of neighbors of the same sex at distance *r*. The bivariate density correlation functions $C_{m1,g2}(r)$ and $C_{m2,g1}(r)$ estimate the correlation between the herbivore-attack intensity of female or male palms, respectively, and the number of neighbors of the opposite sex at distance *r*. The grey dashed lines represent the expected functions of the null models, the dotted lines are the functions for the observed data, and the colored shades show the global simulation envelopes for each type of herbivory. *P* values from the GoF test are shown only for significant effects. Note that Figures S4a-c refer to Figure 3e (female-female), Figures S4d-f to Figure 3f (female-male), Figures S4g-i to Figure 3g (male-male), and Figures S4j-l to Figure 3h (male-female) from the main document.

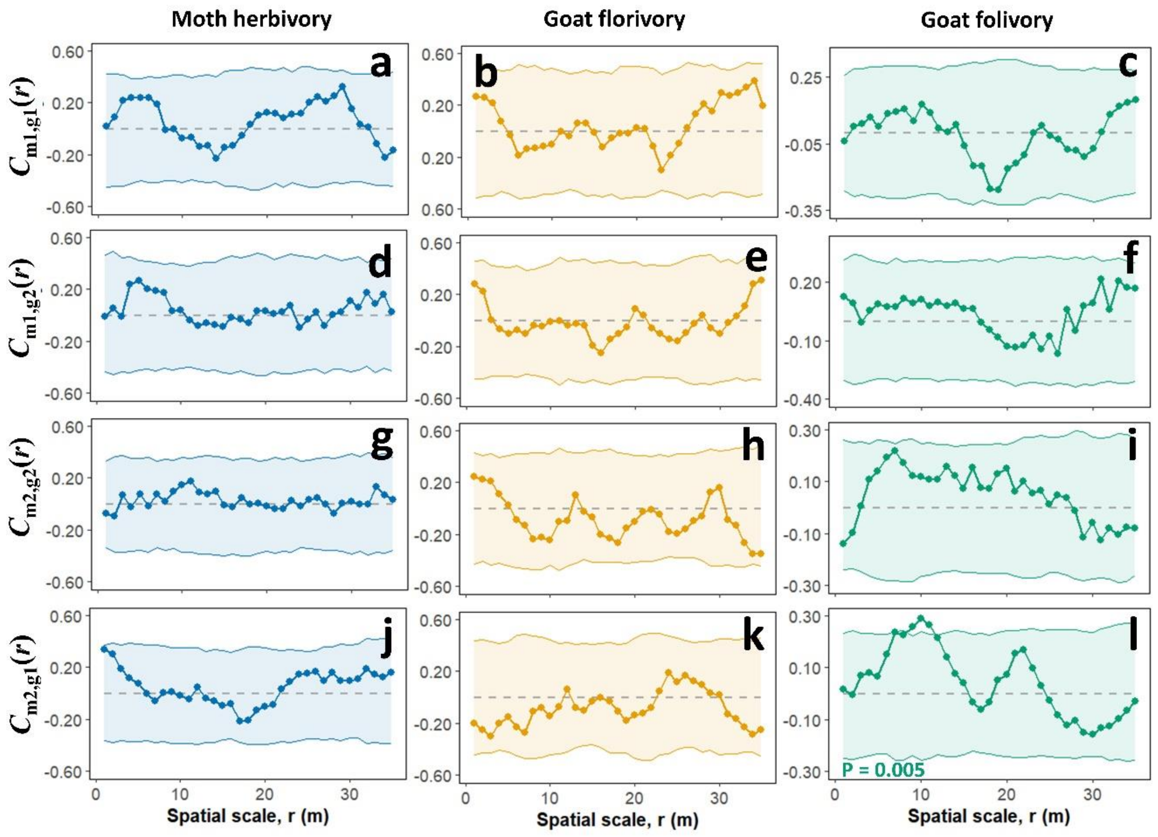

Figure S5. Conspecific neighborhood effects on key plant traits influencing herbivory such as the number of inflorescences (yellow circles) and palm size (i.e. the total number of stems, green triangles) depending on the palm sex (female *vs*. male) in *EB* (a – d) and *PF* (e – h). The univariate density correlation functions $C_{m1,g1}(r)$ and $C_{m2,g2}(r)$ estimate the correlation between the plant trait of female or male palms, respectively, and the number of neighbors of the same sex at distance r. The bivariate density correlation functions $C_{m1,g2}(r)$ and $C_{m2,g1}(r)$ estimate the correlation between the plant trait of female or male palms, respectively, and the number of neighbors of the opposite sex at distance r. The grey dashed lines represent the expected functions of the null models, the dotted lines are the functions for the observed data, and the colored shades show the global simulation envelopes for each plant trait. *P* values from the GoF test are indicated only for significant effects.

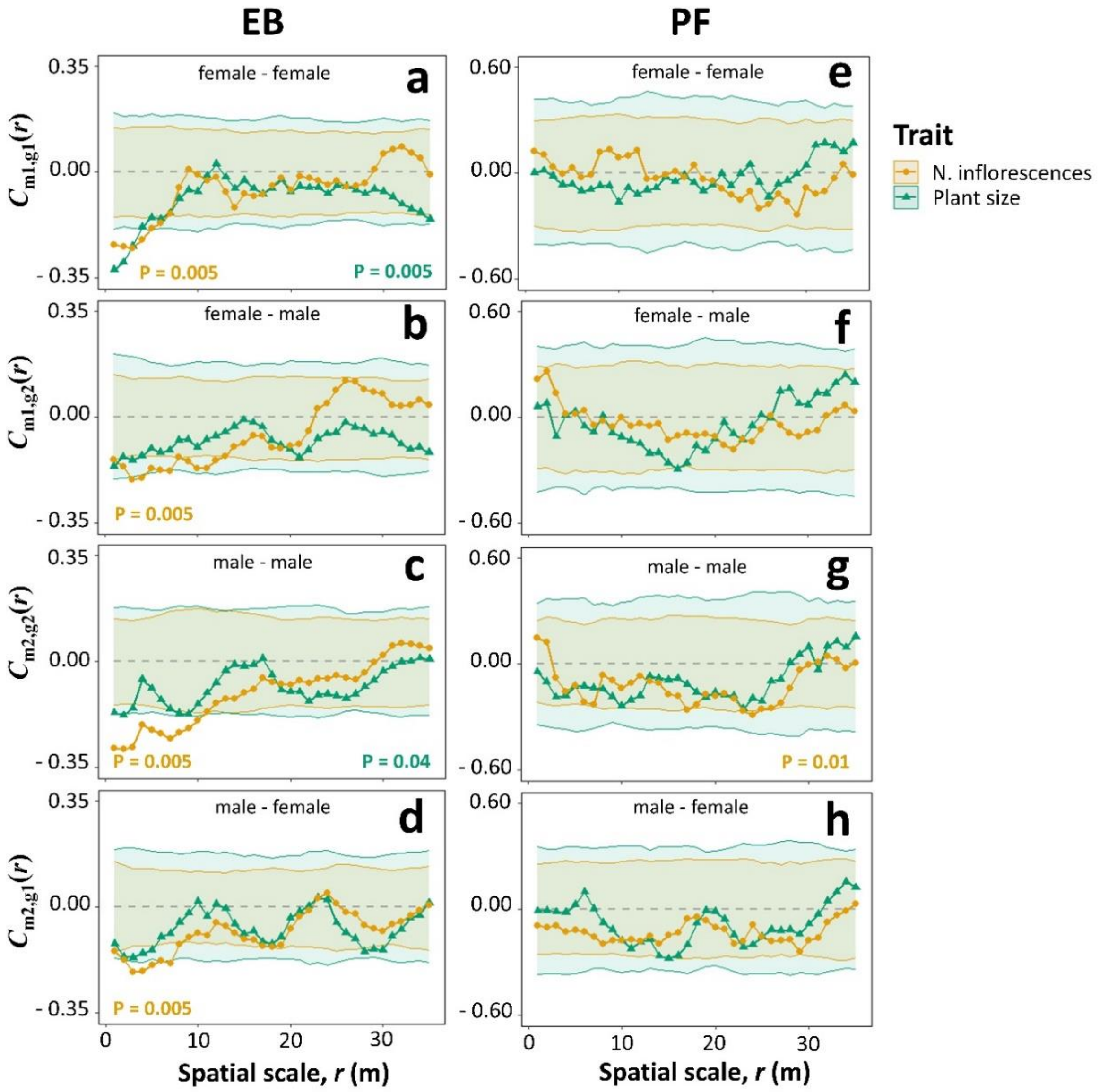

Supplement: Supplementary file 1 — Supplementary file1 (PDF 1197 KB) [file 442_2023_5457_MOESM1_ESM.pdf]
